# Theta burst repetitive transcranial magnetic stimulation attenuates somatosensory evoked potentials from the lower limb

**DOI:** 10.1186/1471-2202-13-133

**Published:** 2012-10-31

**Authors:** Christopher M Zapallow, Michael J Asmussen, David A E Bolton, Kevin G H Lee, Mark F Jacobs, Aimee J Nelson

**Affiliations:** 1 Department of Kinesiology, University of Waterloo, Waterloo, Canada; 2 Department of Kinesiology, McMaster University, Hamilton, Canada

**Keywords:** Continuous theta burst stimulation, Somatosensory evoked potentials, Hoffmann reflex, Lower limb

## Abstract

**Background:**

Continuous theta burst stimulation (cTBS) is a form of repetitive transcranial magnetic stimulation which has been shown to alter cortical excitability in the upper limb representation of primary somatosensory cortex (SI). However, it is unknown whether cTBS modulates cortical excitability within the lower limb representation in SI. The present study investigates the effects of cTBS over the SI lower limb representation on cortical somatosensory evoked potentials (SEPs) and Hoffmann reflex (H-reflex) following tibial nerve stimulation at the knee. SEPs and H-reflex were recorded before and in four time blocks up to 30 minutes following cTBS targeting the lower limb representation within SI.

**Results:**

Following cTBS, the P1-N1 first cortical potential was significantly decreased at 12–16 minutes. CTBS also suppressed the P2-N2 second cortical potential for up to 30 minutes following stimulation. The H-reflex remained statistically unchanged following cTBS although there was a modest suppression observed.

**Conclusion:**

We conclude that cTBS decreases cortical excitability of the lower limb representation of SI as evidenced by suppressed SEP amplitude. The duration and magnitude of the cTBS after effects are similar to those observed in upper limb studies.

## Background

Decades of research in animal models have formulated principles that underlie plasticity within the primary somatosensory cortex (SI) [1]. In humans, plasticity-inducing transcranial magnetic stimulation (TMS) protocols may be applied over SI to promote rehabilitation following injury [2], investigate somatic influences in motor control [3] and to understand the neural basis of touch perception [4]. To date, studies have focused primarily on altering excitability of upper limb representations within SI. However, investigating the effects of these same protocols on lower limb representations would provide insight into both the generalizability of these plasticity- inducing techniques to other body representations, and potentially lead to the development of new strategies to alter cortical activity in patients with sensory and/or motor impairments affecting the lower limb.

Continuous theta burst stimulation (cTBS) is a plasticity-inducing protocol that alters activity within SI such that somatosensory evoked potentials (SEPs) are decreased [5,6]. Specifically, cTBS applied over SI suppresses low frequency median nerve SEP components for up to 13 minutes [5] or leaves them unchanged [6]. High frequency oscillations are also modified by cTBS over SI such that early and late components are facilitated and suppressed, respectively, for up to 15 minutes following stimulation [6]. Of the plasticity-inducing protocols, cTBS offers the advantage of requiring short stimulation durations (i.e. 40 seconds) and relatively long-lasting effects [7]. Although the mechanisms of cTBS effects are not fully understood, cTBS appears to alter inhibitory and excitatory circuitry such that GABAergic [8,9], glutamatergic [10,11] and dopaminergic [12] systems are altered following stimulation. Studies in rat models reveal that TBS alters inhibitory neuronal circuitry within targeted cortex [13,14]. In support of these findings, cTBS increases GABA concentration within human cortex as measured using magnetic resonance spectroscopy [9]. However, NMDA receptor blockade eliminates the effects of cTBS thereby suggesting that glutamatergic systems are also influenced by cTBS [10,11]. Similarly, blockade of the dopamine D2 receptor eliminates the TBS effects [12].

To date, cTBS protocols have aimed to alter the excitability of upper limb representations within SI [5,6] and M1 [5,7,15]. However, there exist distinct differences between hand and lower limb which may cause cTBS induced effects to differ between skin surfaces. Within SI of non-human primates, the hand representation exhibits a much larger cortical territory and higher cortical magnification factor than the lower limb [16]. The lower limb representation is intersected by the foot representation, resulting in two discrete foci dedicated to the lower limb, a trait not seen in the upper limb and hand [16,17]. Last, the glabrous skin of the hand has a propensity for inducing experience-dependent plasticity within SI [18,19] whereas hairy skin, as seen in the lower limb, does not [20]. Further evidence is also found in humans such that a greater number of cortical synapses are involved in the processing of upper limb versus lower limb afferent input [21] thereby increasing the opportunity to modify afferent processing originating from the upper limb. Last, plasticity induced by the lower limb is not identical to that induced by other non-upper limb areas, such as the face. Following rTMS over the leg area, the motor output of ischemic-nerve-blocked upper limb was unchanged, whereas rTMS over the face resulted in long lasting decreases [22]. The aforementioned findings lead to the suggestion that the hand and upper limb may be targeted and modulated via cTBS protocols to a greater extent and with greater ease compared to the lower limb.

The goal of the present study was to investigate the effects of cTBS applied over the lower limb representation of SI on cortical excitability as measured with SEPs. The ability to modify cortical excitability within lower limb representations creates new opportunities for developing therapeutic strategies in clinical populations who demonstrate impairments in lower limb control. Further, it remains to be seen whether cTBS effects on upper limb SEPs may be generalized to lower limb representations. To address these questions SEPs were recorded before and for up to 30 minutes following cTBS over the lower limb representation within SI. Further, we obtained measurements of Hoffman reflexes (H-reflexes) in soleus muscle to identify whether cTBS induced effects occur at the cortical and/or spinal level. Based on the timeline of cTBS effects on early median nerve SEPs [5] it was hypothesized that a decrease in the first tibial nerve cortical potential would be seen up for up to 13 minutes following stimulation.

## Methods

### Participants

Nineteen healthy participants were tested (14 male, mean age = 25.1, SD = 5.24). Thirteen individuals participated in Experiment 1 (10 male, mean age = 25.2, SD = 5.0), six in control Experiment 2 (4 male, mean age = 20.5, SD = 1.22), and three in control Experiment 3 (3 male, mean age = 25.7, SD = 3.06). Three individuals participated in both Experiments 1 and 3 (3 male, mean age = 25.7, SD = 3.06). All participants were right handed, as determined by the Waterloo Handedness questionnaire, a derivative of the Edinburgh Handedness Inventory [23]. All participants wore 30 dB earplugs throughout all experimental procedures. All participants provided informed written consent prior to the experiment. This study conformed to the Declaration of Helsinki and was approved by the University of Waterloo.

### Electromyography (EMG)

Muscle activity was recorded over the right soleus (SOL) and first dorsal interosseous (FDI) muscles using 9 mm Ag-AgCl surface electrodes. For SOL, two electrodes were positioned approximately 2 cm apart over the muscle belly. For FDI, the active electrode was placed over the muscle belly while the reference electrode was placed over the metacarpophalangeal joint of the index finger. EMG was amplified 1000x and filtered from 20–2500 Hz using an Intronix Model 2024F isolated preamplifier (Intronix Technologies Corporation, Bolton, Canada) and acquired using Signal Software and a Cambridge Electronic device (Power 1401, Cambridge Electronic Design, Cambridge, UK).

### Somatosensory evoked potentials

The tibial nerve was stimulated (square wave pulse, 0.5 ms duration, 0.5 Hz) at the popliteal fossa (Grass SD 9, Grass Technologies, West Warwick, USA) with the anode of the stimulating electrode placed approximately 2 cm proximal to the popliteal crease, and the cathode proximal to the anode, to elicit an H-reflex in SOL [24]. Nerve stimulation intensity was maintained at an intensity to evoke an M wave of 10% of the maximal M wave. The 10% M wave intensity was monitored and maintained throughout the experiment.

**Figure 1:**
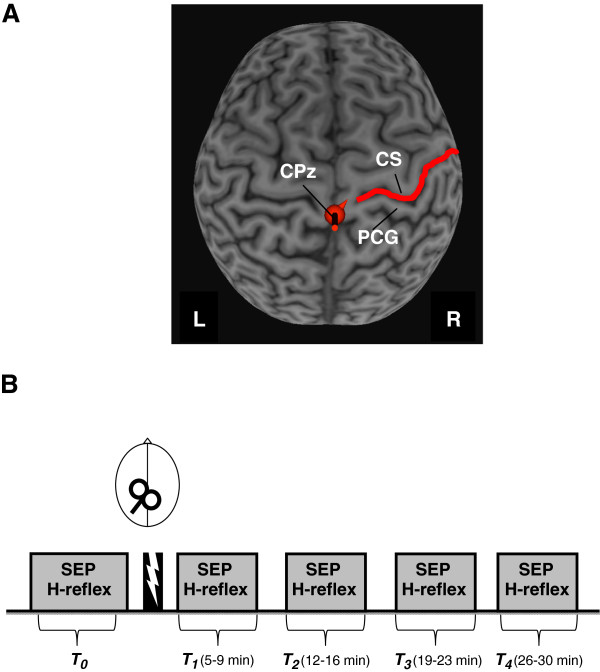
**A. Depiction of localization of cTBS target over CPz.** CTBS was applied over the CPz location which was also the site for recording SEPs following tibial nerve stimulation. CS (central sulcus), PCG (postcentral gyrus). **B.** Experiment timeline. SEPs and H-reflexes were recorded before (*T*_*0*_) and at 5 to 9 (*T*_*1*_), 12 to 16 (*T*_*2*_), 19 to 23 (*T*_*3*_) and 26 to 30 (*T*_*4*_) minutes following SI cTBS.

SEPs were recorded from CPz located over the somatosensory vertex and referenced to FPz [25] with a clavicle ground. In 8 participants, we were able to additionally obtain a MRI to confirm the location of CPz over SI using Brainsight Neuronavigation Software (Rogue Research, Montreal, Canada), as shown in Figure 1A. MRI was collected with a 3 Tesla GE scanner (172 images) with 3DFSPGR-IR sequences using a 20 cm FOV (256x256). EEG recordings were amplified 10000x and filtered from 2–500 Hz (Intronix 2024F Isolated Preamplifier, Intronix Technologies Corporation, Bolton, Canada). Electrode impedances were maintained at <5kΩ throughout the experiment (UFI Checktrode, Model 1089 Mk III, UFI, Morro Bay, California, USA). SEPs were collected in epochs of 150 ms including a 30 ms pre-stimulus period. SEPs were averaged from 120 nerve stimuli delivered every 2 seconds. Previous studies examining tibial nerve evoked SEPs used 50 to 80 [26], 100 [27] to 128 [28,29] nerve stimuli. The frequency of stimulation was chosen to ensure H-reflexes were not suppressed.

### Transcranial magnetic stimulation (TMS)

TMS was applied using a MagPro stimulator (MCF-B65, Medtronic, Minneapolis, Minnesota, USA) using a 90mm diameter figure-of-eight coil. The coil was positioned over left M1 at a 45° angle to the mid-sagittal line to evoke motor evoked potentials (MEPs) in the right FDI. Once an optimal location for FDI MEPs was located, the resting motor threshold (RMT), defined as the lowest intensity required to evoke MEPs of at least 500 μV in 5 of 10 consecutive trials while the muscle is at rest, was found. RMT was determined at the beginning of the experiment prior to nerve stimulation set-up. Continuous theta burst stimulation (cTBS) using 600 pulses [7] was applied over the location of the sensory vertex electrode (CPz). We chose to use a cTBS intensity determined from RMT obtained from FDI based on a pilot study in which we noted that MEPs from the tibialis anterior and soleus muscles were inconsistent, frequently evoked activity in gluteal, hamstrings and quadriceps muscles, and required high intensities (i.e. ~ 85-95% of the MSO) using our figure-of-eight coil which created participant discomfort. Further, cTBS applied at these intensities may result in current spread, making inferences about results due to changes in SI difficult. To improve the opportunity to modify the excitability within the lower limb representation, we subsequently chose to increase the intensity required for RMT and increase the cTBS intensity to 80% RMT, as opposed to the 80% active motor threshold value used in most upper limb studies. Further, we chose to use RMT rather than AMT to avoid metaplasticity effects of cTBS [30] which may contribute to the variable effects of cTBS observed elsewhere [31].

For cTBS, the coil handle was positioned to induce an initial current in the anterior to posterior direction. Prior to application of cTBS, the CPz electrode was removed from the scalp to ensure accurate application of the cTBS and following the completion of cTBS the CPz electrode was replaced at the original location on the scalp and impedances were checked to ensure proper re-application similar to that performed elsewhere [4,32]. This procedure was performed to allow the cTBS coil to be placed directly upon the cranium though we recognize that it may also introduce some variability in the data.

### Experiment 1: Real cTBS over SI

Thirteen subjects participated in Experiment 1. Measures of SEPs and H-reflexes were obtained immediately before cTBS (*T*_*0*_) and at four time blocks following stimulation: 5 to 9 (*T*_*1*_), 12 to 16 (*T*_*2*_), 19 to 23 (*T*_*3*_), and 26 to 30 (*T*_*4*_) minutes. The experiment timeline is shown in Figure 1B.

### Experiment 2: Sham cTBS over SI

Six subjects participated. Experiment 2 was identical to Experiment 1 with the exception that Sham cTBS was applied over SI. For sham stimulation, the real cTBS protocol was delivered with the coil tilted perpendicular to the cranium and positioned over the CPz electrode position. In this orientation, the current flow is mainly directly towards outside of the cranium.

### Experiment 3: Extended duration real cTBS over SI

Three subjects participated. Experiment 3 was identical to Experiment 1 with the exception that three additional time blocks were included to extend the recording window to 60 minutes following cessation of cTBS. These time blocks were 36 to 40 (*T*_*5*_*),* 46 to 50 (*T*_*6*_), and 56 to 60 (*T*_*7*_) minutes following cTBS. Based on the findings of Experiment 1, this control experiment investigated whether P2-N2 changes returned to baseline by 60 minutes following cTBS.

### Data analysis

Two one-way repeated measures ANOVA were performed, one analyzing peak-to-peak amplitude of SEP components and the H-reflex, and a second analyzing onset latency of SEP components and the H-reflex, both with within-subject factor TIME (5 levels: *T*_*0*_*; T*_*1*_*; T*_*2*_*; T*_*3*_*; T*_*4*_). Peak-to-peak SEP amplitudes were measured for the P1-N1 and P2-N2 tibial nerve cortical potentials by averaging 120 epochs during each time block. Trials displaying movement, blink, or noise artifacts were identified and rejected during off-line analysis. A priori hypotheses tested that P1-N1 would be suppressed at *T*_*1*_ (5 to 9 minutes following cTBS) and *T*_*2*_ (12 to 16 minutes following cTBS) as compared to *T*_*0*_. Post-hoc analysis was performed using Tukey’s tests.

## Results

### Experiment 1: Real cTBS over SI

All participants successfully completed the experiment. Data from one individual was removed due to movement related noise in the SEP recordings. Analyses were subsequently performed on data from the remaining 12 participants (10 male, mean age = 25.3, SD = 5.45). The group-averaged RMT and cTBS intensity (with standard deviation) were 55 ± 8.4 % and 44 ± 6.8 % of the maximum stimulator output (MSO), respectively. The latencies of the P1, N1, P2, N2 SEP components and H-reflex (with standard deviation) for each time block are shown in Table 1. With the exception of P2, the latencies of all components remained unchanged following cTBS (P1: F_(4,44)_=0.71, p=0.59, N1: F_(4,44)_=0.68, p=0.61, P2: F_(4,44)_=3.36, p=0.017, N2: F_(4,44)_=0.99, p=0.42, H-reflex: F_(4,44)_=0.0, p=1.0). Post hoc Tukey’s test revealed that the latency of the P2 component was significantly altered at T_2_, T_3_, and T_4_ compared to T_0._ Therefore, the P2-N2 amplitude was analyzed two ways. Method one used the P2-N2 amplitude derived by the latency values obtained from the pre-cTBS block. Method two used the P2-N2 amplitude defined by the P2 latency from each time block separately (i.e. from T_0_, T_1_….).

**Table 1:** Group-average latency of SEP components and H-reflex (mean ± SD)

	**P1**	**N1**	**P2**	**N2**	**H-reflex**
T0	30(2.8)	40(2.8)	47(2.9)	70(7.3)	30(1.5)
T1	31(3.6)	41(4.3)	48(4.3)	67(7.4)	30(1.7)
T2	32(2.9)	42(2.9)	51(3.8)	69(6.2)	30(1.7)
T3	31(3.2)	42(3.7)	49(3.7)	69(6.7)	30(1.7)
T4	31(2.5)	41(4.2)	49(3.9)	69(7.5)	30(1.7)

### Somatosensory Evoked Potentials

**Figure 2:**
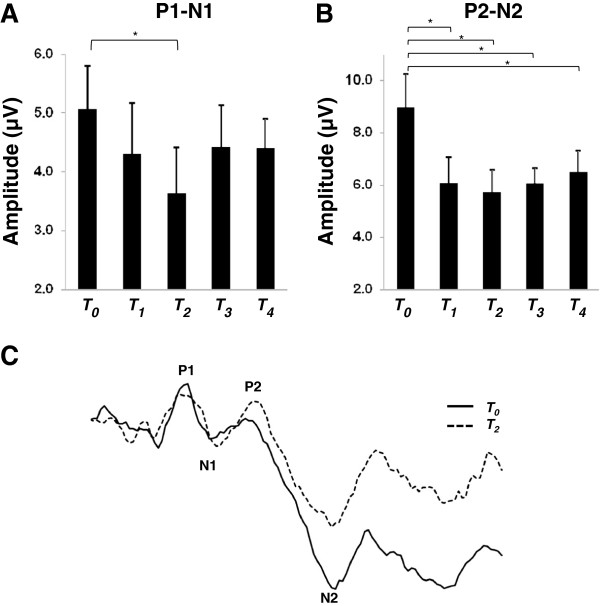
**SEPs before and after cTBS. A**. Group-averaged P1-N1 (with standard error) recorded before and at each time block following cTBS. **B**. Group-averaged P2-N2 (with standard error) recorded before and at each time block following cTBS. **C**. SEP trace from an individual participant depicting location of the P1, N1, P2 and N2 components at *T*_*0*_ (before) and at time block *T*_*2*_ (12 to 16 minutes) following cTBS. Asterisks indicate p < 0.05.

Figure 2A displays the P1-N1 group-averaged peak-to-peak amplitude with standard error bars. One-way ANOVA revealed no effect of TIME (F_(4,44)_=1.53, p=0.21). However, in support of the hypothesis, a priori comparison of pre-cTBS amplitude versus T_2_ revealed that P1-N1 is significantly suppressed from 12 to 16 minutes following cTBS (paired *t*-test, p=0.023) and this effect was observed in 9 of 12 participants. P1-N1 suppression was also nearly significant at T_1_ from 5 to 9 minutes following cTBS (paired *t*-test, p=0.03).

Figure 2B displays the group-averaged P2-N2 amplitude with standard error bars. One-way ANOVA revealed a significant main effect of TIME for both analyses (Method one: F_(4,44)_=9.81, p=<0.0001, Method two: F_(4,44)_=3.72, p=0.01). Post-hoc Tukey’s tests revealed that P2-N2 was significantly suppressed at each time block compared to pre-cTBS amplitude using Method one and at *T*_*2*_, *T*_*3*_ and *T*_*4*_ using Method two.

### H-reflex

**Figure 3:**
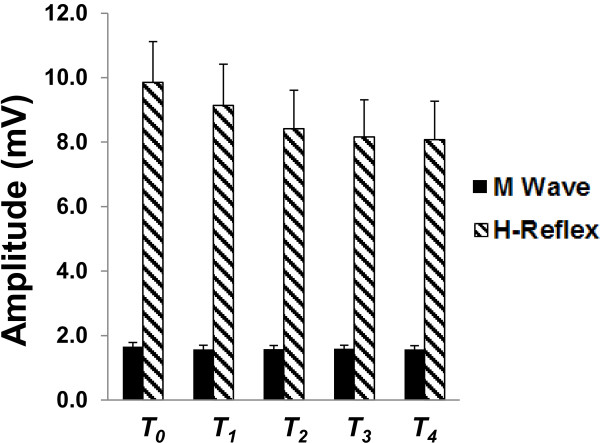
**Group averaged H-reflex and M wave amplitude (with standard error)**.

Figure 3 displays group-averaged H-reflex and M wave amplitudes with standard error for each time block. For the H-reflex, one-way ANOVA revealed no effect of TIME (F_(4,44)_=1.77, p=0.15) suggesting that cTBS over SI did not significantly alter spinal motor neuron excitability. Similarly, for the M wave, one-way ANOVA revealed no effect of TIME (F_(4,44)_=0.56, p=0.69) indicating that nerve stimulation intensity was maintained across time blocks.

### Experiment 2: Sham cTBS over SI

**Figure 4:**
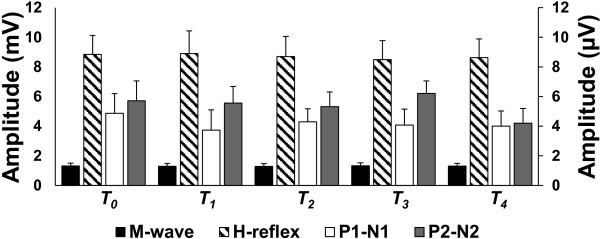
**Sham cTBS over SI. Group averaged peak-to-peak amplitudes (with standard error) of SEPs and spinal reflexes before and at each time block following cTBS.** No significance was found for any measure at any time point. The left ordinate reflects the M wave and H reflex and the right ordinate scale reflects the SEPs.

All participants successfully completed the experiment. Data from one individual was removed due to excessively large potentials deemed to be statistical outliers in the pre-cTBS condition. Analyses were subsequently performed on data from the remaining 5 participants (3 male, mean age = 20.4, SD = 1.34). The group-averaged RMT and cTBS intensity (with standard deviation) were 51 ± 8.9 % and 41 ± 7.3 % of the MSO respectively. Figure 4 displays the group-averaged M wave, H-reflex, P1-N1, and P2-N2 peak-to-peak amplitudes (with standard error) for each time block. One-way ANOVA revealed no effect of TIME for M wave (F_(4 16)_=0.48, p=0.75), H-reflex (F_(4 16)_=0.36, p=0.83), P1-N1 (F_(4 16)_=1.52, p=0.24), or P2-N2 (F_(4 16)_=1.97, p=0.15) suggesting that sham cTBS had no significant effect on either cortical or spinal excitability.

### Experiment 3: Extended duration real cTBS over SI

**Figure 5:**
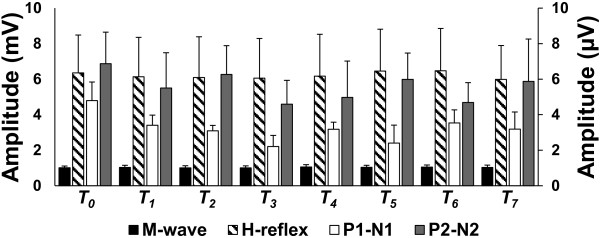
**Extended duration real cTBS over SI. Group averaged peak-to-peak amplitudes (with standard error) of SEPs and spinal reflexes before and at each time block following cTBS.** Three additional time blocks were added to extend the recording window to 60 minutes following cTBS. The left ordinate reflects the M wave and H reflex and the right ordinate scale reflects the SEPs.

All participants successfully completed the experiment. The group-averaged RMT and cTBS intensity (with standard deviation) were 56 ± 15.1 % and 45 ± 12.1 % of the MSO respectively. Figure 5 displays the group-averaged M wave, H-reflex, P1-N1, and P2-N2 peak-to-peak amplitudes (with standard error) for each time block. The data suggest that the P2-N2 amplitude returns to baseline by 60 minutes following stimulation.

## Discussion

In the present study we investigated the effects of cTBS over the SI representation of the lower limb on somatosensory evoked potentials and spinal reflexes originating from tibial nerve stimulation. Novel findings include suppression of the P1-N1 component from 12–16 minutes and reduced P2-N2 for up to 30 minutes following cTBS. CTBS did not significantly alter spinal H-reflexes. These results indicate that cTBS exerts similar effects on lower and upper limb SEPs and that cTBS can be applied over SI to modulate the excitability of the lower limb representation.

Early cortical SEP potentials following median nerve stimulation are thought to originate from the arrival of the thalamo-cortical volley from the thalamus into area 3b of SI [33] and a similar path is postulated for early cortical potentials following stimulation of the tibial nerve [34,35]. The P1 is generated by depolarization of the apical dendritic membrane of pyramidal neurons in the cortex, resulting in a relative electronegativity in reference to the cell soma and basal dendrites [36]. As this current propagates towards the soma and basal dendrites a dipole is formed with the negative pole being located at the apical dendrites (the “sink”) and the positive pole being located at the basal dendrites (the “source”) [36]. This dipole is recorded via EEG as a positivity as the electrode only records from the “visible” side of the dipole, with the negative dipole being occluded [36]. Conversely, the N1 results from the dipole generated by the same population of pyramidal cells with the “sink” being located at the basal dendrites, and the “source” being located at the apical dendrites [36]. The amplitude of these potentials reflects the magnitude of the summation of extracellular postsynaptic potentials created by the depolarization of the pyramidal neurons located at the generator site and the resultant dipole [36]. A suppression of the P1-N1 therefore reflects decreased depolarization of cortical pyramidal neurons and therefore decreased activity within this neuronal population.

The finding that tibial nerve P1-N1 SEPs are suppressed following cTBS closely matches results obtained from a study examining median nerve SEPs. Tibial nerve P1-N1 is suppressed for up to 16 minutes following cTBS but is not suppressed by 19 minutes. Median nerve P25-N33 potentials are suppressed for up to 13 minutes but are unchanged when tested at 20 minutes [5]. Further, the magnitude of cTBS induced suppression is similar for the lower and upper limb. CTBS decreases tibial nerve P1-N1 by ~28% while median nerve P25-N33 is reduced by ~25% [5] or remains unchanged [6]. Comparison of the upper and lower limb data suggests that the time course and magnitude of changes induced by cTBS are similar for the first cortical potential for nerves of the upper and lower limb.

The generator of the tibial nerve derived P2-N2 is still largely unknown. However, somatosensory evoked fields following tibial nerve stimulation suggest that potentials occurring at approximately 50 ms arise from a generator located in area 1 of SI [35]. Further, the P2-N2 appears to originate from a similar large-diameter afferent source that may traverse a polysynaptic pathway before terminating within SI [37]. Similar to the P1-N1, P2-N2 decreased ~ 36% with the maximal suppression occurring from 12 to 16 minutes following cTBS. However, unlike the earlier potential, P2-N2 was significantly decreased for up to 30 minutes with the suppression maintained at ~30%. The robust effects of cTBS on P2-N2 compared to P1-N1 may relate to the spatial proximity between their respective generators and the position of cTBS delivery within SI. Area 1, where later occurring SEPs are thought to be generated, is located on the crown of the postcentral gyrus in close proximity to the focus of cTBS stimulation [38]. In comparison, area 3b, where early cortical potentials are generated, is located in the depths of the posterior bank of the central sulcus [38]. We also observed that the P2 latency increased at 12–16 minutes following cTBS which may also relate to the proximity of the cTBS delivery to the P2 generator. An alternative explanation is that cTBS alters activity in remote loci involved in the polysynaptic pathway through which the P2 travels before arriving in SI [37]. The latter explanation is plausible as cTBS is known to alter activity in remote loci [39].

The H-reflex remained statically unchanged both in latency and amplitude following cTBS. This agrees with previous work showing that cTBS over M1 did not alter H-reflexes [7] or F-waves following Brodmann area 5 stimulation [40]. However, we note that the H-reflex did show modest suppression beginning at ~ 12–16 minutes following cTBS. Specifically, the H-reflex was suppressed by ~ 15% from the pre-cTBS amplitude. This suggests that effects induced by cTBS over SI are mediated primarily via changes in the excitability of cortical neurons but may also include modest changes in the spinal motor neuron output. Intermittent TBS delivered for five consecutive days over M1 alters H-reflexes in patients with multiple sclerosis [41]. It may be that repeat sessions of cTBS over SI and/or M1 may exhibit changes in spinal motor neuron excitability.

## Conclusion

The results of the present study indicate that cTBS may be used over SI to modify the excitability of the lower limb representation. These findings create new opportunities to investigate the utility of cTBS to modulate SI activity in clinical populations. Understanding the modulation of SI lower limb representations has importance in designing rehabilitation strategies for individuals with impaired gait or balance and/or altered somatosensory processing of the lower limb such as in stroke populations.

## Competing interests

The authors declare that they have no competing interests.

## Authors’ contributions

CMZ conceived of the study, carried out the data collection, analyzed all data, and drafted the manuscript. DAEB assisted with the study design, data collection and editing of the manuscript. MJA, KGHL, and MFJ assisted with data collection, and aided in editing of the manuscript. AJN conceived of the study, assisted with data analysis and assisted with manuscript preparation. All authors read and approved the final manuscript.
